# Synthesis and enhanced humidity detection response of nanoscale Au-particle-decorated ZnS spheres

**DOI:** 10.1186/1556-276X-9-647

**Published:** 2014-11-30

**Authors:** Yuan-Chang Liang, Shang-Luen Liu

**Affiliations:** 1 Institute of Materials Engineering, National Taiwan Ocean University, Keelung 20224, Taiwan

**Keywords:** Structure, Nanoparticle, Surface modification, Sulfide

## Abstract

We successfully prepared Au-nanoparticle-decorated ZnS (ZnS-Au) spheres by sputtering Au ultrathin films on surfaces of hydrothermally synthesized ZnS spheres and subsequently postannealed the samples in a high-vacuum atmosphere. The Au nanoparticles were distributed on ZnS surfaces without substantial aggregation. The Au nanoparticle diameter range was 5 to 10 nm. Structural information showed that the surface of the annealed ZnS-Au spheres became more irregular and rough. A humidity sensor constructed using the Au-nanoparticle-decorated ZnS spheres demonstrated a substantially improved response to the cyclic change in humidity from 11% relative humidity (RH) to 33% to 95% RH at room temperature. The improved response was associated with the enhanced efficiency of water molecule adsorption onto the surfaces of the ZnS because of the surface modification of the ZnS spheres through noble-metal nanoparticle decoration.

## Background

Semiconducting compounds have been proposed as potential materials for use in sensing devices for gas detection and humidity measurement [1-4]. In particular, because of their high surface-to-volume ratio, nanostructured semiconductors exhibit physical and chemical behaviors that are superior to their bulk counterparts [5-7]. Among various sensors, humidity sensors have crucial applications in semiconductor electronics and food-processing industries. Various semiconducting materials have been used in humidity-sensing devices [8,9]. The ZnS-based humidity sensors have been realized through complex processes or a high-temperature process [10,11]. Humidity sensors based on ZnS with a facile synthesis methodology are limited in number. ZnS is one of the most crucial II to VI semiconductor compounds [12]. ZnS has shown promise for use in novel diverse applications including light-emitting diodes, sensors, infrared windows, electroluminescent materials, and flat-panel displays [3,12-15]. ZnS nanostructures can be synthesized by various physical and chemical methodologies [3,16]. Although thermal evaporation has been widely used for synthesizing nanoscale ZnS, both the extremely high process temperature and complex process control prevent the realization of high-performance ZnS-based sensors [16]. From a morphological perspective, enhancing the sensing performance of nanostructures continues to be challenging, despite their sensing properties being superior to those of thin films and bulk materials. Recently, surface functionalization with noble metals or through noble-metal doping has been achieved, and it enhances the sensitivity and stability of nanostructure-based sensors [17-19]. However, most of these studies have focused on gas-sensing behaviors, and there are few reports on the humidity-sensing behavior. Recently, Pd^2+^-doped ZnO nanotetrapods were prepared and the humidity detection capability of ZnO was improved through noble-metal doping [20]. Room-temperature humidity-sensing properties of boron nitride nanotubes have been enhanced through surface decoration with Au particles [21]. In this study, a combination of a chemical solution process and the sputtering technique was used to prepare Au-nanoparticle-decorated ZnS spheres. The effects of the surface modification of ZnS-based humidity sensors through Au-nanoparticle decoration were investigated in this study. The ZnS-based humidity sensor performance was observed to be correlated with microstructural changes.

## Methods

The zinc nitrate (Zn(NO_3_)_2_ · 6H_2_O) and thioacetamide (TAA) were used as source materials to prepare hydrothermally synthesized ZnS spheres in this work [22]. The polyvinylpyrrolidone (PVP) was used as a surfactant to control the ZnS sphere size. The 200-nm-thick SiO_2_/Si (100) substrates were used as templates for deposition of ZnS spheres. The reaction solution contains equimolar of zinc nitrate (0.05 M) and TAA (0.05 M). The PVP was subsequently added to the above solution. The reaction solution was stirred at room temperature for 30 min. Subsequently, the reaction solutions and the substrates were transferred into a Teflon-lined stainless steel autoclave. The hydrothermal reaction temperature was fixed at 130°C and the duration is 6 h. At the end of the growth period, the substrates were removed from the solution, then immediately rinsed with deionized water to remove any residual salt from the surface, and dried in air. For synthesis of Au-nanoparticle-decorated ZnS spheres, Au ultrathin film was deposited onto the surfaces of the hydrothermally synthesized ZnS spheres using a home-built DC sputtering system. During deposition, substrate temperature was maintained at room temperature, and the deposition gas pressure was fixed at 20 mTorr with a pure Ar ambient. The sputtering time and power for the Au are 40 s and 20 W, respectively. The samples were further annealed in a high vacuum chamber (base pressure approximately 3 × 10^−6^ Torr) at 300°C for 30 min to induce ultra-thin Au film to form Au nanoparticles on the ZnS surfaces (ZnS-Au). The 200-nm-thick SiO_2_ layer herein was used as an insulator layer. The ZnS and ZnS-Au spheres were dispersed onto the SiO_2_ layer. Subsequently, the silver glue was used to fabricate two metal electrodes onto the ZnS/SiO_2_ for electric measurements.

Crystal structures of the samples were investigated by X-ray diffraction (XRD; Panalytical X’Pert Pro MPD) using Cu K_α_ radiation. Morphologies of the as-synthesized samples were characterized by scanning electron microscopy (SEM; Hitachi S-4800), and high-resolution transmittance electron microscopy (HRTEM; Philips Tecnai F20 G2) was used to investigate the coverage and morphology of Au nanoparticles on the surfaces of the ZnS spheres. The energy-dispersive X-ray spectroscopy (EDS) attached to TEM was used to evaluate the composition of the samples. Room temperature-dependent photoluminescence (PL; JOBIN-YVON T64000 Micro-PL Spectroscopy) spectra were obtained using the 325-nm line of a He-Cd laser. The electrical characteristics of the ZnS-based sensors were tested as a function of relative humidity (RH) with a fixed applied voltage of 5 V in a home-built testing chamber at room temperature. A computer was used to collect the signals from the sensor in the testing circuit. The RH levels for the humidity sensor test herein were controlled to be approximately 11%, 33%, 55%, 75%, 85%, and 95% and a hygrometer was used to monitor RH levels in the test chamber. The experimental setup has been described elsewhere [23]. The different RH levels were generated by referencing various saturated salt solutions in closed chamber at room temperature [24]. The humidity sensitivity test of the samples was performed with the sample initially stored in the dry ambient (11% RH); subsequently, the sensor was upon exposure to one of the higher selected RH levels (33% to 95%). Finally, the sensor was restored in the 11% RH environment again to finish a test run.

## Results and discussion

**Figure 1:**
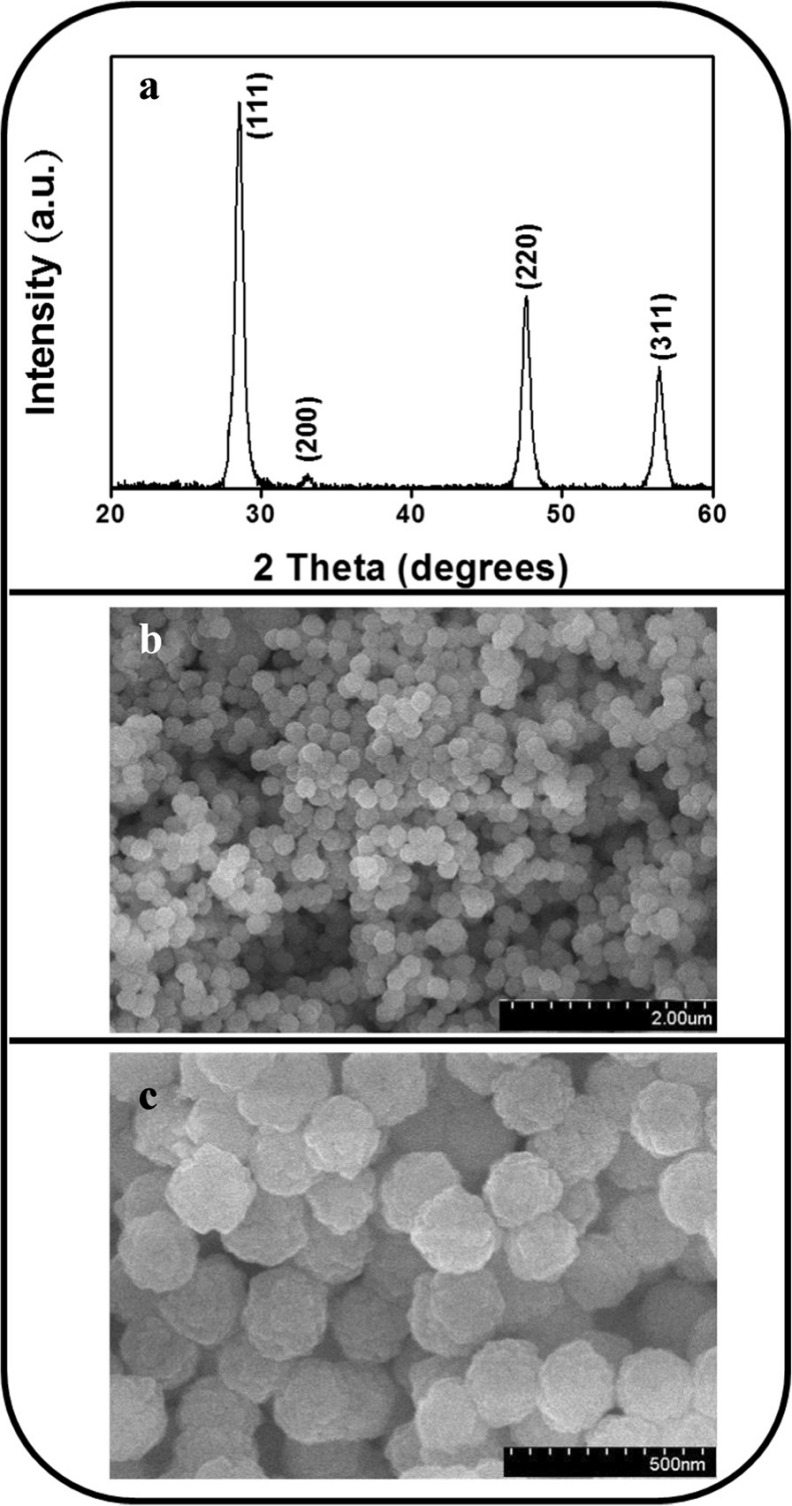
**XRD pattern of the ZnS-Au spheres and SEM micrographs of the ZnS spheres. (a)** XRD pattern of the ZnS-Au spheres. **(b)** Low-magnification SEM micrograph of the ZnS spheres. **(c)** High-magnification SEM micrograph of the ZnS spheres.

Figure 1a shows an XRD pattern of the ZnS-Au spheres, which reveals several Bragg reflections from the 111, 200, 220, and 311 planes of the cubic ZnS structure (JCPDS No. 05-0566). No clear Au signals were detected; this might be because the Au particles are small and content is not large enough to contribute marked diffraction signals. The XRD patterns show that highly crystalline ZnS spheres were synthesized. Figure 1b,c presents SEM micrographs of the synthesized ZnS spheres. The monodispersed ZnS spheres homogeneously covered the substrate. The surface of the ZnS spheres was irregular. The monodispersed ZnS sphere had a diameter of approximately 200 nm. In the Au-nanoparticle-decorated ZnS spheres (data not shown here), the SEM micrographs provided insufficient information to distinguish the change in surface features of the ZnS spheres following Au decoration. Detailed TEM studies, discussed in the next section, have further elucidated the microstructure of the Au-nanoparticle-decorated ZnS spheres.

**Figure 2:**
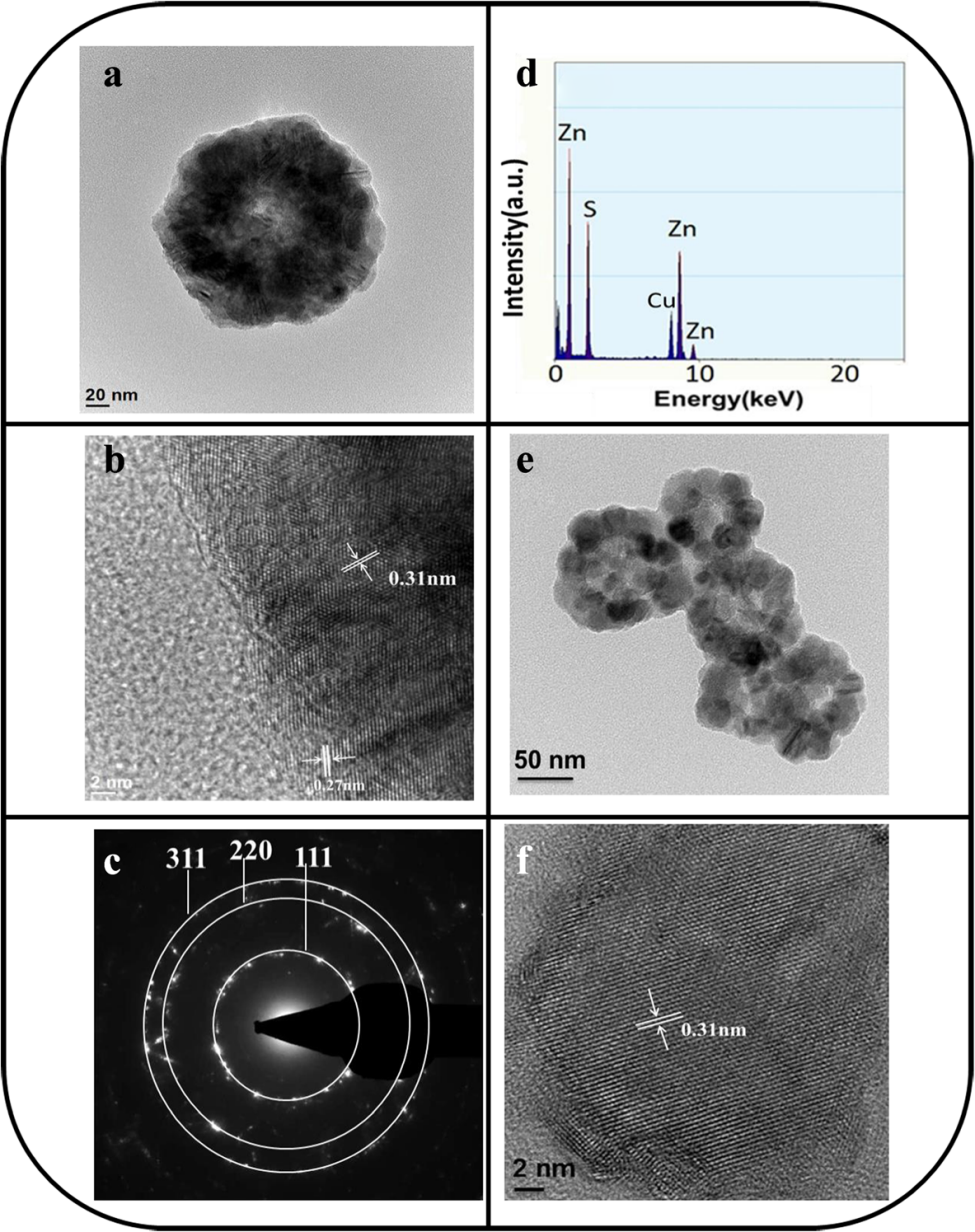
**TEM analyses of the ZnS spheres. (a)** Low-magnification TEM image of a ZnS sphere. **(b)** HRTEM image taken from the local region of the ZnS sphere. **(c)** SAED pattern of the ZnS sphere. **(d)** EDS spectrum of the ZnS sphere. **(e)** Low-magnification TEM image of the ZnS subspheres. **(f)** HRTEM image taken from the local region of the ZnS subsphere.

**Figure 3:**
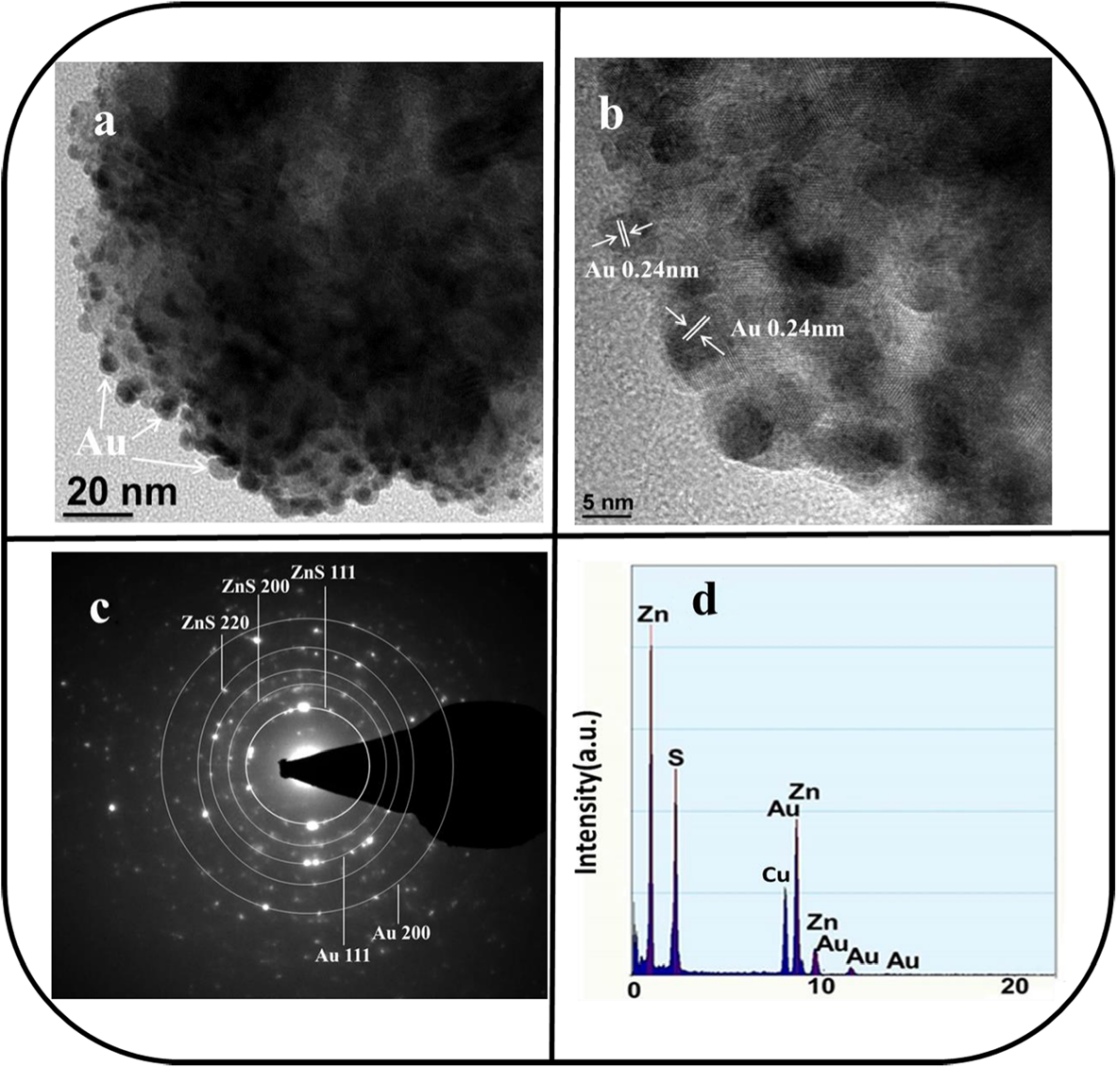
**TEM analyses of the ZnS-Au spheres. (a)** Low-magnification TEM image of a ZnS-Au sphere. **(b)** HRTEM image taken from the local region of the ZnS-Au sphere. **(c)** SAED pattern of the ZnS-Au sphere. **(d)** EDS spectrum of the ZnS-Au sphere.

Figure 2a shows a low-magnification TEM image of a single ZnS sphere prepared in this study. The TEM image further indicates that the sphere has a rough surface, which is in agreement with the observations made from SEM micrographs. The ZnS sphere is composed of many tiny ZnS crystals that are densely packed. The HRTEM image taken from the outer region of the ZnS sphere reveals clear lattice fringes in the ZnS sphere (Figure 2b). Lattice spacings of 0.31 and 0.27 nm are marked in the HR image, and they are assigned to the {111} and {200} planes of the cubic ZnS phase, respectively. The selected-area electron diffraction (SAED) pattern of a ZnS sphere shows several clear diffraction rings that correspond to the cubic ZnS structure, and the ZnS sphere shows polycrystalline characteristics (Figure 2c). The energy-dispersive X-ray spectroscopy (EDS) spectrum of a ZnS sphere reveals that the sphere consists only of Zn and S; no other impurity is observed (Figure 2d). A TEM image of a ZnS sphere that collapsed during the preparation of the TEM sample was used to understand the formation mechanism of the ZnS sphere through hydrothermal synthesis. In Figure 2e, ZnS subspheres clearly have a diameter of approximately 50 to 60 nm. The contrast in the TEM image reveals that these subspheres are composed of many ZnS nanoparticles. These nanoparticles have a diameter of approximately 10 to 20 nm. A typical HRTEM image of a ZnS nanoparticle is shown in Figure 2f. Clear and ordered lattice fringes in the HR image reveal that each ZnS nanoparticle has a single-crystal structure. In this study, the formation of a ZnS sphere through hydrothermal synthesis was associated with the time-dependent clustering of the ZnS nanoparticles [22].Figure 3a shows a TEM image of a ZnS-Au sphere. The Au metallic particles exhibit a dark contrast in the TEM image. Most Au nanoparticles dispersed on the surface of the ZnS and partial Au nanoparticles were mounted on the surface layer of the ZnS. No evidence of large aggregations of Au nanoparticles is apparent. These Au nanoparticles rendered the surface of ZnS porous. Overall, the ZnS-Au sphere surface is more rough and irregular than the pure ZnS sphere surface. An HR image of a part of the surface of a ZnS-Au sphere reveals that the Au nanoparticle sizes are approximately 5 to 10 nm (Figure 3b). A clear lattice spacing of 0.24 nm is observed, and this value is assigned to the {111} plane of Au. Figure 3c shows a SAED pattern of the ZnS-Au sphere. In addition to the diffraction rings originating from the ZnS phase, several rings from the polycrystalline Au phase are also observed. An EDS spectrum of the ZnS-Au sphere revealed that the sample contents were Zn, Au, and S, confirming that Au nanoparticles decorated the ZnS sphere (Figure 3d). The Au-nanoparticle-decorated ZnS spheres had an Au content of approximately 17.5 wt.%. TEM analyses revealed that ZnS spheres that have Au metallic nanoparticles decorating their surface can be obtained using a combination of sputtering and high-vacuum postannealing processes.

**Figure 4:**
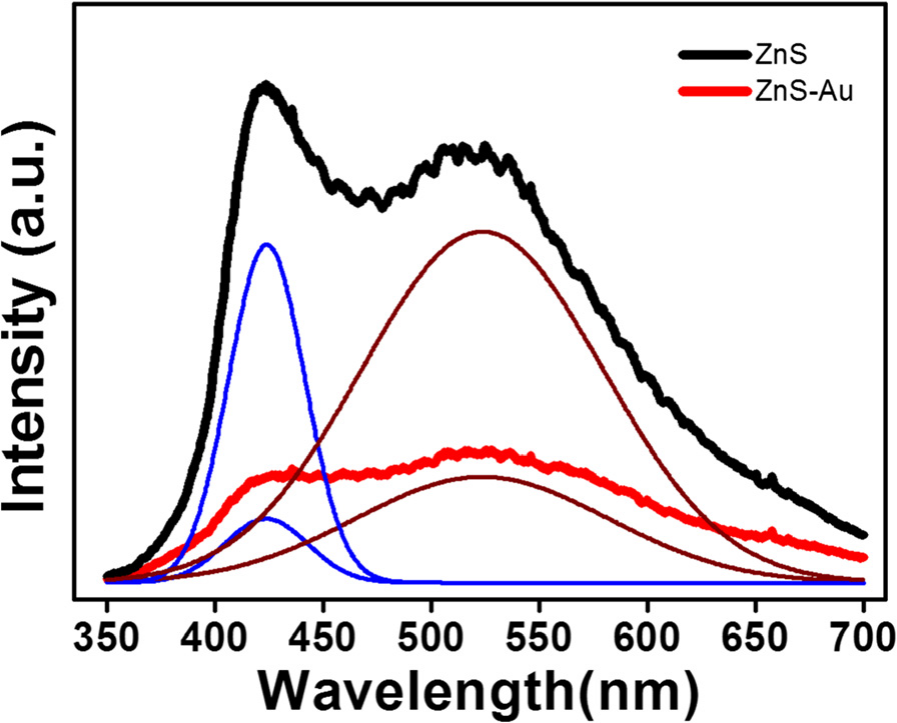
**PL spectra of the ZnS and ZnS-Au spheres.** Each PL spectrum was divided by two Gaussian deconvolution curves which were centered at approximately 425 nm (blue line) and 525 nm (brown line).

Figure 4 shows PL spectra of ZnS spheres with and without Au nanoparticle decoration. Two emission band features can be observed: a blue emission band centered at approximately 425 nm and a green emission band centered at approximately 525 nm. Moreover, two identical emission band features were observed for the ZnS spheres with Au-nanoparticle decoration; however, total spectral intensity and the intensity ratio of the blue emission band to the green emission band were lower. A similar blue emission band has been reported in ZnS with various morphologies. Generally, the blue emission band is associated with the existence of crystal defects such as sulfur vacancies, interstitial sulfur, and zinc vacancies [4,25]. The green emission band has been associated with the surface states of ZnS [26,27]. However, a similar green emission that has been reported in the Au-catalyzed growth of ZnS nanowires is caused by the substitution of Zn ions by Au ions in the ZnS lattices during high-temperature processes [28]. Such substitution cannot explain the observed green emission band of pure ZnS spheres in the present study because no Au was used in the hydrothermal synthesis process. The distribution of Au nanoparticles on the surface of ZnS further quenched the PL spectrum intensity in this study. The plasma-sputtering metal process might have damaged the ZnS surface to a certain extent, and the subsequent high-vacuum annealing process might have induced surface sulfur atoms to fill the lattice vacancies. These effects might account for the difference in intensity ratio of the blue emission band to the green emission band between the PL spectra of ZnS spheres with and without Au-nanoparticle decoration.

**Figure 5:**
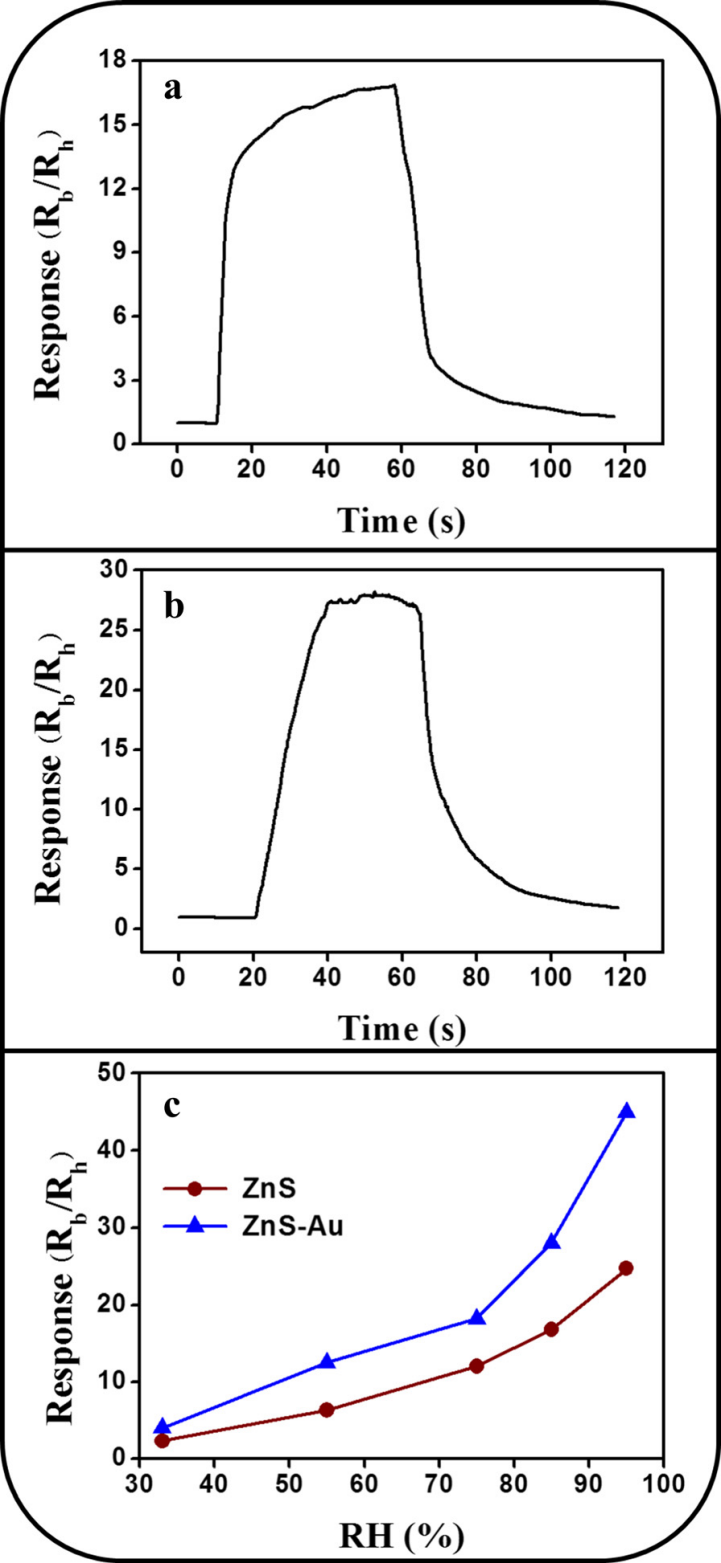
**Humidity-sensing responses of the ZnS and ZnS-Au sensors.** Humidity-sensing responses of the ZnS and ZnS-Au sensors operated at room temperature upon cyclically exposure from 11% RH to 85% RH to 11% RH: **(a)** ZnS spheres, **(b)** ZnS-Au spheres, and **(c)** the summarized response values of the ZnS and ZnS-Au sensors exposed to various RH levels.

The synergistic effect of ZnS spheres decorated with Au nanoparticles on the humidity-sensing performance was further investigated. The resistance variation of ZnS and ZnS-Au sensors was monitored by alternately exposing the sensors to 11% RH (base humidity) and another relative humidity value (33%, 55%, 75%, 85%, or 95% RH). Figure 5a,b shows typical humidity response characteristics of the sensor resistance to the low (11% RH)-high (85% RH)-low (11% RH) cycle. The response was defined as R_b_/R_h_, where R_b_ is the sensor resistance at 11% RH and R_h_ is that at the test RH level. The response of the samples on exposure to 85% RH was approximately 17 and 28 for the ZnS spheres and ZnS-Au spheres, respectively. The sensors showed marked humidity selectivity. The characteristics of the humidity response curve can be explained as follows: in the initial state (sensor was stored in a low-humidity environment; base humidity: 11% RH), the sensor had the highest resistance. Subsequently, when the sensor was exposed to a high-humidity environment (85% RH), the resistance decreased and gradually achieved a relatively stable value. The resistance increased and gradually recovered to the initial stable state after it was switched again to a low-humidity environment (11% RH). Figure 5c summarizes the humidity response values of the ZnS-based sensors with and without Au-nanoparticle decoration for selected percent RH levels ranging from 33% to 95%. The humidity response of the sensor constructed using ZnS spheres demonstrated responses of 2.3 to 24.7 for 33% RH to 95% RH. Moreover, the Au-nanoparticle-functionalized ZnS sensor showed responses of 4.2 to 45.3 for 33% RH to 95% RH. The ZnS-based sensor response increased with the RH level. The reason was that a higher number of water molecules could be adsorbed on the surface of the ZnS spheres for a higher RH level, and a greater amount of charge transfer could occur from the water molecules to the ZnS semiconductor in the test environment; an increase in the charge of ZnS resulted in the lowered resistance of the sensor in relatively high-humidity conditions [24]. Comparatively, the humidity responses of the ZnS spheres were markedly enhanced on Au-nanoparticle decoration. This result indicated the improved degree of resistance variation of the ZnS spheres under the various percent RH levels following Au-nanoparticle decoration. The humidity-sensing behavior is related to water adsorption and desorption processes on the surface of ZnS spheres [29]. Reversible physisorption of water molecules is a strong function and chemisorption of water molecules plays a minor role in the variation of resistance of n-type semiconductors operating in relatively high percent RH levels. The water molecular adsorption rate has been shown to depend on the surface area of the ZnO nanoparticles [30]. The irregular surface of the ZnS spheres, which is associated with the ZnS nanoparticle aggregation, provides several adsorption sites for water molecules. Therefore, marked humidity responses of the ZnS-sphere-based sensors were observed in this study. Oxide microspheres with a porous morphology have been produced previously; such morphology enhances the surface-area-to-volume ratio [31]. In TiO_2_-SnO_2_ thin films, the particle size (which is of the order of nanometers) and the porous surface facilitate the adsorption of water molecules [32]. Structural analyses indicated that ZnS sphere surfaces became rougher and more irregular on Au-nanoparticle decoration, which resulted in a porous surface. With an increasing percent RH level, more water molecules are efficiently intercalated through these porous surfaces. Efficient proton diffusion then occurs through the bonded water molecules. Au-nanoparticle-decorated boron nitride nanotubes exhibit better humidity responses than boron nitride nanotubes without Au-nanoparticle decoration. The presence of Au nanoparticles on the surface of boron nitride nanotubes markedly enhanced the conductance of the semiconductor on exposure to a relatively high-humidity environment [21]. Moreover, the enhanced humidity sensitivity of polyamidoamine dendrimer sensors modified with Au nanoparticles has been reported [33]. In the present study, the enhanced humidity response of the ZnS-Au sensor was associated with the enhanced proton diffusion of the sensor on exposure to high percent RH levels after initial exposure to the base humidity level. This caused a markedly larger variation in the resistance of the ZnS-Au sensor compared with the ZnS sensor.

## Conclusions

Highly crystalline ZnS spheres were decorated with Au particles by combining the sputtering technique and high-vacuum thermal annealing. Detailed TEM images revealed that the as-synthesized Au particles had nanoscale sizes and that they were efficiently distributed on the surface of the ZnS spheres. PL spectra revealed that the nanoparticle surface modification changed the PL spectra intensity and intensity ratio of ZnS emission bands. Au nanoparticles decorating the surface of the ZnS spheres significantly affected the sensor’s humidity response. The ZnS-Au sensor exhibited considerably enhanced sensitivity compared with a pure ZnS sphere sensor at various percent RH levels operated at room temperature.

## Competing interests

The authors declare that they have no competing interests.

## Authors’ contributions

YCL designed the experiments and drafted the manuscript. SLL carried out the sample preparations and the material analyses. Both authors read and approved the final manuscript.

## Authors’ information

YCL is a professor of the Institute of Materials Engineering at National Taiwan Ocean University (Taiwan). SLL is a graduate student of the Institute of Materials Engineering at National Taiwan Ocean University (Taiwan).
